# Prioritization of Economically Important Cattle Diseases Using Participatory Epidemiology Tools in Lalibela, Sekota, and Ziquala Districts of Amhara Region, Northern Ethiopia

**DOI:** 10.1155/2020/5439836

**Published:** 2020-03-01

**Authors:** Adane Bahiru, Ayalew Assefa

**Affiliations:** Sekota Dry Land Agricultural Research Center, P.O. Box 62, Sekota, Ethiopia

## Abstract

Ethiopia's livestock resource is one of the largest globally. It is estimated at around 59.5 million cattle, about 30.5 million sheep, and 30.2 million goats. The sector is irreplaceable in the means of livelihood of the population as a source of meat, milk, drought power, and income. Yet, the country is unable to exploit the sector entirely because of highly prevalent infectious diseases and lack of appropriate disease control policy. These constraints are worse in districts of Lalibela, Sekota, and Ziquala, where this specific study was carried out. Despite the availability of scanty animal health services in these areas, information on animal health, especially cattle, was never a significant focus of research. This study was conducted with the objectives of identifying and prioritizing primary cattle disease with the aid of participatory epidemiology tools. Focus group discussions (FGD) and questionnaires were used in prioritizing the top economically important cattle diseases of the selected areas. Accordingly, the result of FGD indicated that ectoparasites, CBPP, FMD, blackleg, bloody diarrhea, and pasteurellosis were the major diseases affecting cattle production in the area. These areas can be representative of most of the countries where a mixed farming system is practiced. Therefore, this result can be used as a basis for broader planning of prevention and control strategies for these kinds of diseases. However, a laboratory-supported extensive investigation of these diseases is highly recommended to validate findings of such types of prioritization of diseases.

## 1. Introduction

Ethiopia's livestock resource is one of the largest globally. It is estimated at around 59.5 million cattle, about 30.5 million sheep, and 30.2 million goats [1]. The country's economy mostly depends on livestock next to crop production. Moreover, the sector is irreplaceable in the livelihood of the population as a source of meat, milk, drought power, and income. However, despite having this massive amount of resource, the country is unable to exploit the sector entirely because of highly prevalent infectious diseases and lack of appropriate disease control policy [2].

Waghemira zone (where two of the three study districts are found) has a total of 375,878 cattle, 216,029 sheep, 418,022 goat population, and 66,401 beehives. Even though there is an ample amount of livestock resources in the area, livestock diseases and the prevailing drought mostly remain the significant constraints limiting the sector's productivity. High animal mortality and impaired fertility rates contribute to poverty and increase their vulnerability. The disease prevalence is high due to environmental factors like high temperature and weak animal health services [1].

In areas like these study districts where animal health facilities and professionals are lacking, participatory epidemiology (PE) tools play a significant role in profiling important health constraints of the sector. The expanding use of PE demonstrated the flexibility of participatory methods and the relevance of the methods in resource-poor settings. After many years of decline in government veterinary services in developing countries, PE seemed to play an essential role in helping researchers and government epidemiologists to reconnect with livestock keepers and gain a better understanding of diseases from a local perspective. What is far less evident is the extent to which these activities led to improved disease control, with related benefits for people. Other than the successes of the global rinderpest eradication campaign where PE contributed to programs in East Africa and Pakistan, and some CAHW programs, there are few examples in the literature where research or surveillance using PE resulted in more successful disease control [3].

Despite the use of some scanty veterinary services, information on animal health, especially cattle, was never a significant focus of research in this area. However, knowing the type and extent of the common and significant health problems of cattle in the area is very important for veterinarians, researchers, and other responsible governmental and nongovernmental bodies. These kinds of health information can assist in the development of herd health strategies and the selections of possible intervention approaches. By understanding this, we designed participatory profiling and prioritization of economically significant diseases of cattle using participatory epidemiology tools in the three selected districts.

## 2. Materials and Methods

### 2.1. Description of the Study Area

**Figure 1:**
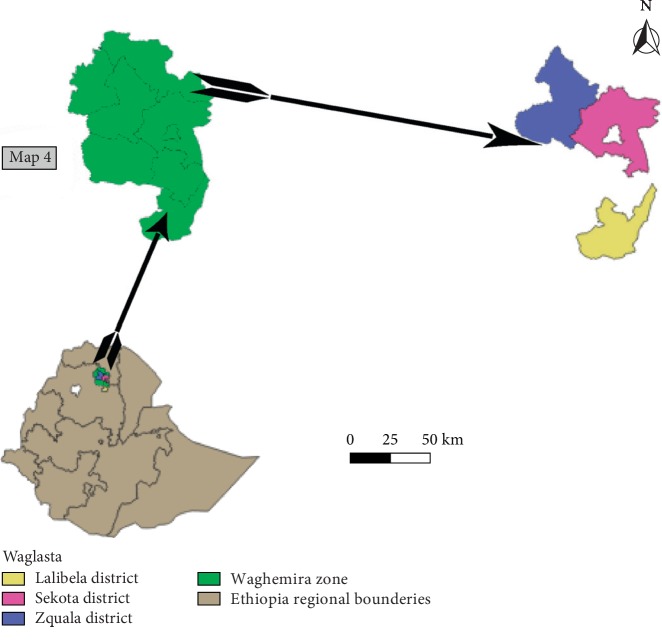
Map of study districts.

The study was carried out in three selected districts (Ziquala, Sekota, and Lalibela) of Amhara regional state of Northern Ethiopia (Figure 1). Study sites were selected based on an agroecological difference to represent lowland and highlands. Ziquala district is found in the Waghemira zone of the Amhara region and agroecologically characterized as hot, warm submoist lowland located at an average altitude of 1450 masl. Lalibela district, on the other hand, is found in the North Wollo zone of the Amhara region located in latitude coordinates of 12°01′60″ North and longitude coordinate of 39°01′60″ East. The average elevation of the district ranges from 1600 to 4200 masl. Sekota district is also found in the Waghemira administrative zone of the Amhara region located between 12°23′ and 130°16′ north longitudes and 38°44′ and 39°21′ east latitudes. The district represents hot to warm submoist agroecology having an altitude of 1800–2200 masl [4].

### 2.2. Study Design and Sampling Strategy

A cross-sectional study was conducted from June 2016 to December 2018 from the three districts. Study sites were selected by a stratified sampling approach. Strata were made based on the agroecological difference of the administrative zone. Households from each district were selected randomly for the focus group discussion (FGD). Study animals were also selected by a simple random sampling technique.

### 2.3. Focus Group Discussion (FGD)

The participatory epidemiology (PE) method was used in each study district to get prior information about significant cattle disease of the study area. A total of 10–15 participants were selected from each district based on their livestock production status and knowledge of essential livestock diseases. Participant groups included elders, youth, men, and women to represent all gender groups by introducing PE tools like interviews, ranking, and scoring methods. During FGDs, participants discussed the importance of different livestock species and allocated 100 counters across the species mentioned to indicate their relative importance. In the second part, the participants listed five crucial cattle diseases that affected their herds and described the clinical signs of these diseases, and they were requested to distribute 100 counters to indicate the relative importance of these diseases. Participants were also asked to list the primary season of disease occurrence in their locality and conduct proportional piling of the ‘important counters' of each disease across the identified seasons. Besides, participants conducted proportional piling of the ‘important counters' of each disease across age and sex categories of animals to assess which group of animals are affected by a disease. The last part of the FGD focused on disease transmission. Participants were asked to explain and list up to five possible disease transmission pathways. Proportional piling was done by using 20 counters per transmission pathway/situation to find out who (men, women, young men, young women, and children) is mainly involved in specific transmission situations.

### 2.4. Questionnaire Survey

A semistructured questionnaire was administered to a total of 31 randomly selected household heads from all the three districts. Questionnaires were developed with the main focus on cattle health constraints, husbandry practices, feed, and feeding systems.

### 2.5. Data Analysis

The collected data were stored in a Microsoft Office Excel 2016 spreadsheet and analyzed descriptively.

## 3. Results and Discussion

### 3.1. Demographic Characteristics of Participants

The age of the participants ranges from 25 to 75, with a mean age of 45.79 years. Most of the participants were unable to read and write (52.6%) and the remaining have completed primary school (21.1%), can read and write (21.1%), and completed secondary education (5.3%). This is due to the low coverage of education in the area compared to another part of the country [4]. Most of the interviewed individuals were males (63.2%), whereas 36.8% were females. It is because most of the agricultural practices are under the command of men in Ethiopia as well as this specific study site.

The majority of the participants herd their cattle alone (94.7%), and the remaining 5.3% herd their cattle together with other species of animals. Participants claimed that they feed their animals in groups (36.8%) and separately (63.2%). The majority (63.2%) claimed that they migrate their animals for grazing at a distance of <5 km, while the rest (31.6% and 5.3%) move their cattle more than ten and >5 km, respectively.

Most of the participants (68.4%) have access to veterinary services at a distance less than 5 km, and the remaining 31.6% get service at a distance greater than 5 km. The level of satisfaction with the vet service they get was high (52.6%), moderate (36.8%), and low (10.5%). Participants indicated that the reasons for little satisfaction include no reason to describe (47.4%), unavailability of qualified experts (31.6%), the high price of the vaccines and drugs (15.8%), and unavailability vaccine and medications (5.3%).

According to participants, the most susceptible animals from the herd are calves, followed by cows and bulls. This is, in fact, true that newborns are mostly sensitive to infectious diseases due to weak immunity level and failure to get a vaccination at early age because in the Ethiopian farming system, there is a tendency not to vaccinate younger animals. Besides, according to interviewers, the most important diseases of the area were ectoparasites and respiratory disease followed by unknown diseases with sudden death. This result somehow agrees with the findings of the FGD.

### 3.2. Findings of the FGD

Upon prioritization of the five major cattle diseases in the study districts, they gave priority to ectoparasites, which were prioritized to be the most important animal health challenges in the study area. Next to ectoparasites, farmers prioritized contagious bovine pleuropneumonia (CBPP). It is a respiratory disease complex with a higher fatality rate and low vaccination coverage in the area. The other most crucial diseases raised by participants were foot and mouth disease (FMD), blackleg, bloody diarrhea, and pasteurellosis which are mentioned as the diseases found in the study area. This result is in line with many pieces of research conducted throughout the country. In almost all parts of the country, ectoparasites have been reported as highly prevalent with huge economic loss [5]. Besides, infectious diseases like blackleg, FMD, and CBPP have been reported widely [6–10]. The overall score for the prioritization of disease scores have been depicted in the Table 1. The score for some diseases like diarrhea and pasteurellosis was zero, while these diseases were mentioned in Sekota and Ziquala districts which were piled together.

The main routes and people involved in disease transmission were market route (men) (44/100), herd contact (youth male) (12/100), common grazing land (youth male) (24/100), and communal watering routes (youth male) (22/100). The clinical signs described for most of the diseases raised by farmers (ectoparasites, CBPP, FMD, blackleg, diarrhea, and pasteurellosis) were consistent with clinical symptoms and indicators described in the veterinary literature and textbooks. However, a laboratory-supported wide investigation of these diseases is highly recommended to validate findings of such types of FGDs.

Livestock species prioritization indicated that groups from the Lalibela district, cattle (54/100), take the highest share of their livelihood, followed by sheep and goat (24/100), honey bee (11/100), and equine (11/100), respectively. However, in Sekota and Ziquala, sheep and goat (45/100) take the highest share, followed by honeybee (22/100), cattle (18/100), and equine (12/100). Participants from the Lalibela district reported that cattle have higher importance than other livestock species. This can be due to the fact that the area is found above midlatitude with a relatively suitable environment for cattle production than the other study districts. On the other hand, participants from the Sekota and Ziquala districts reported that sheep and goats have a higher advantage than other livestock populations. These districts are semiarid with the harsh environment in which Abergelle breed goats were found predominantly than other livestock species. It might not be surprising if they said these species are more important than other species because the area is not ideal for cattle production.

Furthermore, participants discussed that market and communal grazing are the main routes of disease transmission. These areas are very prone to animal mix up that can lead to imminent disease transmission to a healthy herd. In these routes (market and communal grazing), men and youth groups reported being involved, respectively. In the study area and other rural households in Ethiopia, men are in selling and buying of animals while the youth group has the responsibility of herding in the field. That is why participants indicated that these groups are the main actors of disease transmission.

## 4. Conclusions

As PE tools are useful approaches in prioritizing economically important livestock diseases in many developing countries, this research comes up with a finding that is indicative where responsible bodies act. Farmers described that ectoparasites, CBPP, FMD, blackleg, and pasteurellosis are the major health constraints in the study areas. These areas can be representative of most of the countries where the mixed farming system is being practiced. Therefore, this result can be used as a basis for broader planning of prevention and control strategies for these diseases.

## Figures and Tables

**Table 1:** Disease priority scores in the study districts.

Disease	Score	
	Lalibela/(100)	Sekota and Ziquala/(100)
Ectoparasites	50	49
CBPP	24	24
FMD	10	13
Blackleg	16	—
Diarrhea	—	10
Pasteurellosis	—	4

## Data Availability

Data will be available upon request.
